# Conversion of Nerves into Fat

**Published:** 1842-10-08

**Authors:** 


*Conversion of Nerves into Fat.*—The body of a male subject, aged 30, was brought for dissection into the anatomical school at Marburg. The whole body was dropsical, and the left leg, from the foot to above the knee-joint, firmly swollen. On the dorsum of the foot were ulcers, from which sinuses could be traced into the tarsal joint. Dissection showed the cellular tissue of the limb infiltrated with plastic lymph, which in the neighbourhood of the ankle had a fatty, and higher up in the limb a fibrous appearance. On account of the carious condition of the joint, as well as the firm nature of this deposit, which was situated between the skin and fascia, and also, beneath the latter, between the muscles, the movements of the lower part of the limb had evidently been suspended for a considerable time. The muscles were pale and flabby, but in other respects not altered in structure. The larger trunks of the nerves in the upper part of the limb were quite normal, but as they approached the affected part they became thickened, and appeared as if composed of mere fat. Portions of the saphenus, and other large branches of the ischiatic, so far as they could be separated from the degenerated mass, with which their sheaths became more and more amalgamated the lower they were traced, were dissected and examined under the microscope; when it was found that an extraordinary quantity of fat had been deposited within the sheath and between the fibres of the nerve, which increased in irregular gradations as it was traced downwards, till it constituted the whole structure of the nerve. The fat globules appeared to be arranged concentrically on the inner surface of the sheath, and by a stronger magnifying power the primitive fibres could, at the upper part, be distinctly seen running in the centre of the fatty deposit. They gradually disappeared lower down, till at length no trace of them could be found, the fat globules having entirely taken the place of the primitive nervous fibres.—*Ibid, from Müller's Archiv.*

